# NMR-Based Metabolomics to Decipher the Molecular Mechanisms in the Action of Gut-Modulating Foods

**DOI:** 10.3390/foods11172707

**Published:** 2022-09-05

**Authors:** Weiwei He, Hanne Christine Bertram

**Affiliations:** Department of Food Science, Aarhus University, Agro Food Park 48, DK-8200 Aarhus, Denmark

**Keywords:** gut-host associations, saccharolytic activity, unfavorable metabolites, calcium

## Abstract

Metabolomics deals with uncovering and characterizing metabolites present in a biological system, and is a leading omics discipline as it provides the nearest link to the biological phenotype. Within food and nutrition, metabolomics applied to fecal samples and bio-fluids has become an important tool to obtain insight into how food and food components may exert gut-modulating effects. This review aims to highlight how nuclear magnetic resonance (NMR)-based metabolomics in food and nutrition science may help us get beyond where we are today in understanding foods’ inherent, or added, biofunctionalities in relation to gut health.

## 1. Introduction

Omics has become a widely used concept in life science and inherently annotates a field of study ending in ’-omics’. The omics concept represents a new way to approach research, where the ultimate aim is a complete and thoroughly characterization a pool of molecules instead of exclusively targeting a narrow research question. Metabolomics, which deals with uncovering and characterizing the pool of metabolites present in a biological system, is now a prime omics discipline [1]. Within food and nutrition, metabolomics has gained wide success with its contribution to deciphering how specific food and food components impact endogenous metabolism in humans [2]. Aligned with an increasing attention to the significant role that the gut microbiome plays in human health, the focus has recently become centered on employing metabolomics to obtain insight into how food and food components may exert gut-modulating effects [3]. Fecal material contains unabsorbed food residues that are directly available to the gut microbial community. Thus, by employing metabolomics analyses on fecal samples, it has become evident that information can be obtained in relation to the presence of food-derived metabolites accessible to the gut microbiome, and the complex interactions between food components and the metabolic activity of the gut microbiota [4,5]. Furthermore, by multiple sampling of content in the gastrointestinal tract, metabolomics can provide comprehensive information concerning bioaccessibility, absorption processes, and escape of food residues into the colon [6]. Studies focused on exploring the capacity of analytical tools for metabolomics research have revealed that more than 70 metabolites can be detected in feces by proton nuclear magnetic resonance (^1^H NMR) spectroscopy [7,8]. The identified metabolites include amino acids and derivatives, organic acids, carbohydrates (mono- and disaccharides), biogenic amines, and nucleotide derivatives (Table 1) as well as other metabolites derived directly from ingested food [7,8]. Moreover, ^1^H NMR spectroscopy is also able to monitor changes in certain gut-derived metabolites (e.g., short-chain fatty acids (SCFAs), amines, and trimethyl amine-N-oxide (TMAO)) in other biofluids such as urine and blood [9,10]. Therefore, through the application of NMR-based metabolomics, it is possible to elucidate the potential molecular mechanisms by which gut-modulating foods affect host health. This review aims to highlight how NMR-based metabolomics in food and nutrition science may aid in understanding foods’ in-build and added biofunctionalities in relation to gut health. This is achieved by presenting examples of different food items. Previous publications have summarized in detail how NMR-based metabolomics works, and we refer to these for further information on technical details in applications of NMR-based metabolomics [11,12].

## 2. Advantages and Limitations of NMR-Based Metabolomics

In the 1980s, NMR was first applied to the analysis of rat urine by Nicholson et al. [13] who later proposed the term metabolomics [14]. Over the past two decades, both NMR-based metabolomics and subsequently developed mass spectrometry (MS)-based metabolomics have been widely used to explore metabolite profiles of bio-samples. As an alternative and complementary method to (MS)-based metabolomics, NMR-based metabolomics has specific advantages, including high reproducibility, less requirements for sample preparation (e.g., fewer preparation steps, time-saving, chromatography-free, and/or derivatization-free), and inherent quantification without the use of specific internal standards. These advantages make NMR-based metabolomics the preferred choice for high-throughput analyses and inter-laboratory comparisons.

As a main limitation, NMR-based metabolomics is characterized by lower sensitivity than MS-based metabolomics [15]. Typically, NMR-based metabolomics can detect up to 50 quantifiable metabolites in bio-samples, whereas MS-based metabolomics can capture in the range of 1000 features. A higher metabolite coverage obviously increases the chances of discovering changes or differences. On the other hand, NMR-based metabolomics is considerably further developed in relation to automation of analyses, and recently an NMR platform for clinical quantitative diagnosis has been launched by the Bruker IVDr platform. In addition, based on the non-invasive feature and the advantages of enabling analysis of solid or semisolid samples, NMR-based metabolomics has the potential to determine metabolite changes in intact tissue samples and in vivo studies without the requirement for metabolite extraction [15,16]. In food and nutrition science, the further development of NMR equipment and NMR metabolomics technologies [15], including increased sensitivity of probes, optimized NMR excitation pulse schemes (e.g., DREAMTIME [17]), hybrid NMR approaches (e.g., liquid chromatography NMR [18]) and faster NMR spectra preprocessing (e.g., SigMa [19]) is considered important.

## 3. Multiple Purposes of NMR-Based Metabolomics in Food and Nutrition Science

**Figure 1:**
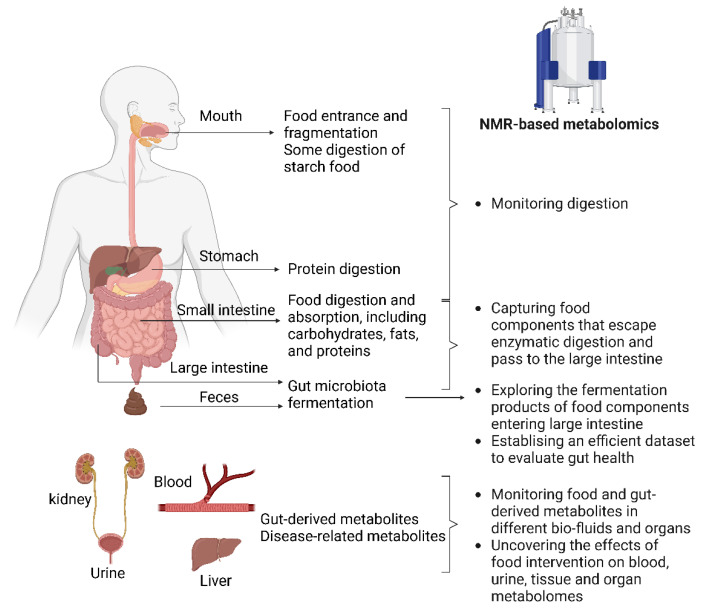
Multiple purposes of NMR-based metabolomics through analysis of samples collected from different sites of gastrointestinal contents, bio-fluids, tissues, and organs. Figure created with Biorender.com.

Analysis of metabolite changes throughout the gastrointestinal tract, and in feces by NMR-based metabolomics, enables elucidation of how food and food components are digested, absorbed, and fermented in the gastrointestinal tract. Furthermore, by adding NMR-based metabolomics analyses on blood, urine, organs and tissues, information can be obtained about effects on the host’s metabolic homeostasis and health (Figure 1).

A high-field NMR instrument allows the acquisition of spectra that can capture changes in amino acid and mono or disaccharides throughout the gastrointestinal tract and in feces, which reflects digestive properties (e.g., protein and saccharides) and the amount of food components that escape digestion in the small intestine and pass to the distal intestine. As an example, by employing NMR-based metabolomics in the analysis of small and large intestinal contents, Lanng et al. [4] observed increased amino acid signals in the small intestine and colon in rats fed with pork partly substituted with insect components compared to rats fed with pork only, indicating that insect proteins may be more likely to escape small intestinal digestion and pass to the colon in healthy rats. In addition, He et al. found that inulin ingestion gave rise to strong inulin signals (fructose and anomeric glucose) in NMR spectra obtained for content of the small intestine, while these signals subsequently gradually disappeared in spectra obtained on content of the large intestine, thereby supporting its digestion by the gut microbiota and prebiotic effects in the distal gut [20].

The application of NMR-based metabolomics on distal gut contents, feces, or in vitro fermentation samples enables determination and characterization of bacterial fermentation products of indigestible food components (e.g., protein and saccharides). Thus, NMR-based metabolomics can provide insight into differences in gut modulatory capacity among various fiber sources. By determining the differences in fermentation products between indigestible saccharides obtained from different sources or with different chemical structures, a strategy can be devised to modulate the generation of short-chain fatty acids (SCFAs) [21,22]. Increasing evidence shows that dietary protein sources may also affect gut microbiota activities [4,23,24]. NMR spectroscopy can capture signals of amino acids and derivatives, branched SCFAs, and biogenic amines, and provides a new and alternative method for evaluating the nutritional value of protein sources (e.g., meat proteins, seafood proteins, insect proteins, and plant proteins). Thus, while traditional methods for evaluating the nutritional value of proteins exclusively analyze absorption from the small intestine, NMR-based metabolomics on contents obtained throughout the gastrointestinal tract allows elucidation of the potential impact on gut microbiota activities. 

Distal gut metabolites may reflect the state of gut and host health and may therefore be used as potential diagnostic biomarkers. Current metabolomic techniques have the potential to establish effective datasets to assess gut health by collecting metabolite differences between subjects with different health statuses [25,26]. However, the large individual variability in gut metabolites [27] is a challenge in the development of reliable diagnostic biomarkers. Therefore, big data with absolute concentrations of metabolites (for interlaboratory comparisons) in the intestinal contents or feces are required to establish reliable datasets. NMR-based metabolomics has advantages in being applicable for high-throughput assays, quantitative, and providing excellent reproducibility. To obtain a standardized assay for the analysis of the fecal metabolome, Bervoets et al. established a protocol for NMR-based metabolomics that enabled interlaboratory comparisons and made it possible to establish criteria for assessing infant gut health [28]. Protocols have also been presented for NMR-based metabolomics of feces from adults [7].

Gut permeability reflecting intestinal barrier function is important for preventing the invasion of harmful substances and microbiota. Alinaghi et al. developed an effective NMR-based technique to evaluate gut permeability in piglets by monitoring the concentrations of non-metabolizable molecules (i.e., lactulose and mannitol) in the blood and urine after an exposure test [29]. By using partial least squares (PLS) regression analysis, this study also observed correlations between gut permeability and the NMR metabolite profiles of blood and urine. 

NMR-based metabolomics can monitor changes in gut bacterial-derived metabolites, such as SCFAs, TMAO, benzoate, hippurate, 3-indoxyl sulfate, dimethylsulfone, di/tri-methylglycine, and trigonelline, in the blood, urine, tissues, and organs [30,31], which provides molecular evidence to understand the gut-organ axis and food-gut-organ associations. As well as gut-derived metabolites, changes in other metabolites or disease biomarkers in the blood, urine, tissues, or organs can also aid to understand how gut-modulating foods affect host homeostasis and disease-related metabolism. In addition, NMR-based metabolomics can characterize non-polar metabolites, which is useful in the evaluation of effects of gut-modulating foods on lipoproteins and lipid metabolites in bio-fluids, tissues, or organs [32,33]. Thus, NMR-based metabolomics might be a rapid method that does not require derivatization, compared to gas chromatography (GC)-MS [34,35]. 

Multivariate data analysis (e.g., principal component analysis (PCA) and partial least squares-discriminant analysis (PLS-DA) are commonly used in metabolomics. However, it is a challenge to integrate and interpret multiple NMR profiles. Generally, correlation analysis and multivariate regression (e.g., PLS and OPLS) are used to establish links between metabolites from different types of bio-samples. However, approaches that are more sophisticated have also been used. Multi-block PCA and ANOVA simultaneous component analysis (ASCA) allows integrative analysis of datasets from multiple compartments [36]. Yde et al. (2014) applied multi-block PCA in the analyses of NMR data obtained from plasma, urine, feces and tissue samples from mice fed with different protein diets to link metabolite profiles of different biofluids/organs [37], and an application of penalized exponential ASCA was demonstrated on NMR profiles of different brain tissues (hypothalamus and midbrain) in piglets [38].

Integrated analysis of metagenomes and metabolomes has the potential to link metabolite changes in the blood, urine, tissues, and organs to modulated gut microbiota [39,40,41], which can provide new insights into how gut-modulating foods affect host health. Multivariate data analysis has been applied to reveal the effects of diet intervention on specific gut microbiota and metabolites. Subsequently, the application of statistical correlation analysis (Pearson or Spearman) can support relationships between altered metabolites and altered gut microbiota/gut metabolites [42,43,44,45]. 

## 4. Metabolomics Reveals the Beneficial Effects of Saccharolytic Activity on Gut-Organ Axis

**Figure 2:**
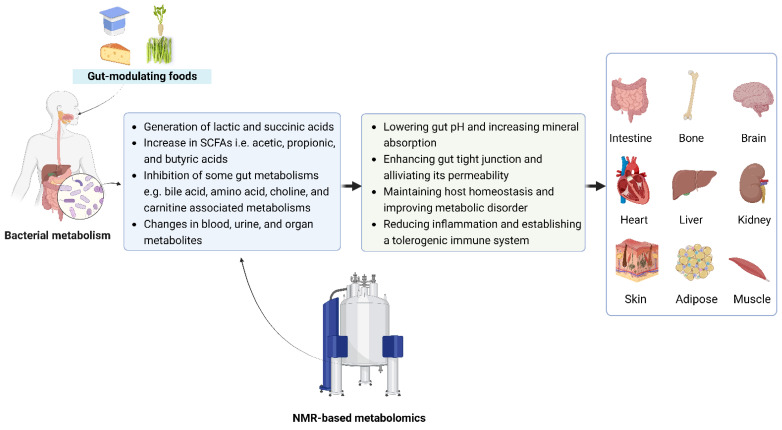
Metabolomics reveals metabolites as potential links between gut-modulating food and different organs, including intestine, bone, brain, heart, liver, kidney, skin, adipose, and muscle [49,50,51]. Figure created with Biorender.com.

One of the powers of metabolomics is the potential to disclose how food and food components modulate metabolic activity in the gut. NMR-based metabolomics can capture and quantify SCFA generation in the gut [20], and increasing evidence suggests that the gut microbiota mediate their beneficial effects through the fermentation of dietary fiber to produce SCFAs, endogenous signals with important roles in metabolic homeostasis and relieving chronic inflammation in humans [46,47]. In addition, SCFAs are also speculated to play a key role in gut-host crosstalk, and the existence of a gut-organ axis has gained attention (Figure 2) [48,49,50,51]. Metabolomics reveals that fiber-derived SCFAs, especially butyrate, are mainly metabolized by colonic epithelium [52,53], promoting the proliferation of epithelial cells, regulating intestinal permeability and enhancing intestinal barrier function [54]. 

Among the potential gut-organ axes, the existence of a gut-bone axis has recently received increasing attention [55]. The gut-bone axis is interesting, since it may unlock how food and food components exert effects on bone health in more ways than by providing calcium and other relevant building bricks of bone tissue. The possible existence of a gut-bone axis investigated in human intervention studies, showing that prebiotics such as inulin and oligosaccharides could promote calcium absorption and bone mineralization in both youth and postmenopausal women [56,57]. Based on this, it has been suggested that metabolic activity in the gut impacts calcium bioavailability. This is a newly discovered field in which metabolomics is a major tool in establishing causal links between functional food, gut, and bone. A rat study using NMR-based metabolomics revealed that inulin supplementation has a strong potential to stimulate the generation of SCFAs, lactic acid, and succinic acid in the gut [20]. Combined with previous evidence that SCFAs could promote calcium absorption [58] and reduce T cell-induced osteoclast formation by establishing a tolerant immune system [59,60,61], underlying mechanisms supporting a link between inulin ingestion and bone mineralization have been established.

Besides samples collected in the gastrointestinal tract, the application of NMR-based metabolomics in the analysis of blood samples has aided elucidation of whether gut-derived SCFAs are directly involved in the regulation of bone homeostasis. Although in vitro studies supported that SCFAs can stimulate the differentiation of bone marrow cells (BMCs), caution should be taken with the idea that gut-derived SCFAs directly participate in the regulation of host homeostasis (e.g., bone metabolism and rheumatoid arthritis) [62,63]. Previous studies have found that increased circulating butyrate and propionate levels obtained by oral supplements of these metabolites were too low to modulate BMC differentiation in mice [63,64]. Thus, the application of metabolomics in the analysis of fecal samples and blood samples enables us to elucidate mechanisms of how gut-modifying food regulates host health.

NMR-based metabolomics also makes it possible to monitor changes in blood and urine metabolites following ingestion of gut-modulated foods in patients with metabolic diseases, which may provide insights for carrying out in-depth mechanistic exploration. Studies have shown that dietary fibers have the potential to prevent overweight and obesity, and the main effects of fiber intake involve increased bacterial saccharolytic activity and SCFA generation in the distal gut [30]. However, the association between gut SCFAs and obesity remains controversial. Although many studies link fiber-induced SCFA generation in the distal gut to the prevention of obesity [30], in a previous analysis higher levels of SCFA were observed in overweight and obese compared to normal weight subjects [65,66,67,68]. Furthermore, conflicting or controversial results were observed in previous studies on how SCFA interventions prevent obesity [69,70]. As well as SCFA generation, fiber saccharolytic activity in the distal gut shapes the gut microbiota and promotes the proliferation of some favorable bacteria such as bifidobacteria, thereby alleviating obesity by the regulation of pathways involved in inflammation, the expression of bile acid receptors, and choline availability [71]. Emerging evidence suggests that changes in blood metabolites are closely related to gut microbiota composition [72] and thus may provide new insights into explaining the fiber-gut-obesity association. 

The application of NMR-based metabolomics in the analysis of blood provides a footprint of whole-body metabolic processes [73], offering opportunities to clarify how fiber intake affects host metabolism in obesity. A prospective study recently discovered that fiber supplementation for 8 weeks reduced blood pressure and increased blood high-density lipoproteins (HDL) in overweight and obese hypertensive women [74]. Furthermore, NMR-based metabolomics revealed that fiber supplementation also increased levels of choline, phosphatidylcholine, and β-hydroxybutyrate, accompanied by a reduction in blood pressure and an increase in HDL. Several other preclinical and randomized controlled clinical trials using NMR-based metabolomics in the analysis of blood metabolites have shown that the beneficial effects of gut-modulating foods (i.e., fiber and probiotics) in body weight management also involve changes in circulating amino acids, glucose metabolites (e.g., pyruvate and succinate), creatine, and creatinine [75,76]. Subsequently, linking changes in circulating metabolites to metabolic diseases is thought to explain the underlying mechanisms. Choline and phosphatidylcholine are important in the synthesis of very low-density lipoproteins (VLDL), thereby participating in the regulation of lipid metabolism in obesity [77,78]. Circulating levels of β-hydroxybutyrate, creatine, and creatinine are associated with muscle mitochondrial respiration and have the potential to regulate adipose tissue metabolism [79,80]. Studies have shown that probiotic intake-induced improvements in adipose tissue metabolism may involve changes in circulating amino acids, although the link between gut microbiota, circulating amino acids, and obesity remains unknown [76]. 

Of note, metabolomics only provides potential associations between metabolites and host health, which implies numerous vulnerable correlations between metabolites and metabolic diseases as they may not reflect causality. Therefore, in-depth studies for revealing the changed metabolism and the underlying causal relationships are important for understanding how gut-regulating foods affect host health.

## 5. Gut Modulating Foods in the Suppression of Unfavorable Metabolites

NMR-based metabolomics provides a tool to unveil how gut-modulating foods (e.g., prebiotics and dietary fibers) affect the generation of some unfavorable gut-derived metabolites such as TMAO. Epidemiological data point at negative effects of red and processed meat intake on colon and host health, potentially associated with the microbiota-derived unfavorable metabolites [81]. However, metabolomics research has revealed that associations between meat intake and formation of unfavorable metabolites are complex and dependent on meal/diet composition. Thus, a study was conducted in which pigs in a two-by-two factorial design were fed with two different meat sources and two different background diets. The two meat sources included either white meat (poultry) or a combination of red and processed meat, while the two background diets consisted of a typical Western background diet with a high content of fat and refined sugars, or a prudent background diet with a high content of vegetables and a low content of fat and refined sugars [10]. Intriguingly, the urine metabolome revealed that while no effect of background diet was seen when white meat was ingested, the background diet had a significant effect on TMAO excreted in urine when red and processed meat was ingested [10]. Thus, when red and processed meat were ingested together with a prudent background diet, the TMAO concentration in urine was similar to that seen after chicken intake, but when red and processed meat were ingested together with a Western-type background diet, the TMAO concentration in urine was significantly increased. The reason that these findings have received attention can be ascribed to the fact that TMAO proposed to link diet with cardiovascular disease risk [82]. The formation of TMAO originates from the gut microbiota’s metabolization of carnitine present in red meat to trimethylamine (TMA), which subsequently can undergo oxidation to TMAO in the liver [83]. By contrast, a high intake of dietary fibers is associated with beneficial effects on colon health [84]. Therefore there has been a growing interest in determining if combining meat with dietary fiber intake could counteract the meat-associated negative effects on colon health [85,86]. Employing an authentic food product solution, a metabolomics study investigated how fortification of a traditional pork sausage product with prebiotic inulin affected the biochemical processes in the gastrointestinal tract of healthy rats ingesting the processed meat products [9]. Compellingly, the metabolomics data revealed a pronounced and significant effect of inulin fortification on SCFAs in the gut. Concomitantly, the blood metabolome revealed that the enhanced formation of SCFAs also resulted in an increased circulating level of acetate [9]. As the study was conducted in healthy rats, it was not possible to validate that the enhanced formation of SCFAs could prevent carcinogenesis; however, examination of the formation of nitroso compounds in the gastrointestinal tract revealed that inulin in fact attenuated formation of nitroso compounds [87].

## 6. Calcium as a New Functional Food Ingredient?

By monitoring gut-derived metabolites, NMR-based metabolomics can be used to explore food components that exert gut-modulating effects. Intriguingly, metabolomics studies have revealed that calcium exerts biofunctional effects in the gastrointestinal tract. Through the application of NMR-based metabolomics analyses on intestinal content, an intervention study with rats revealed that the beneficial effects of calcium supplementation on the proliferation of *Acinetobacter* and *Propionibacterium* were associated with an increased generation of SCFAs in the cecum and colon [20]. Moreover, another NMR-based metabolomics study conducted in rats where the effects of calcium fortification of processed meat products were investigated similarly demonstrated that intake of calcium-fortified meat products for 4 weeks increased the concentration of SCFAs in the gut content when compared with intake of non-fortified meat products [5]. Fuhren and coworkers also observed that calcium phosphate (dairy calcium) supplementation beneficially stimulated the production of SCFAs in the gut, especially combined with inulin and galacto-oligosaccharides [88], indicating calcium supplementation may benefit gut saccharolytic activity. However, the exact mechanisms by which calcium promotes the formation of SCFAs in the gut remain unknown, but the current findings from metabolomics research indicate that the presence of calcium changes the biochemical environment in the gut and thereby favors the presence of specific bacterial species. Consequently, metabolomics studies have supported the evidence that calcium acts as a functional food ingredient with gut-modulating capabilities [85], which might be the potential mechanism by which calcium is beneficial against metabolic diseases such as obesity and lipid dyslipidemia [89]. 

## 7. Conclusions and Perspectives

As a functional read-out of the metabolic activity of the gut microbiome, fecal metabolomics has become a valuable tool for examining how food and food components may exert effects in the gut. SCFAs are considered, if not the most important, then clearly among the chief metabolites that are modifiable by functional foods with a substantial impact on gut health. It is therefore intriguing that NMR-based metabolomics on feces firmly allows the detection and quantification of SCFAs. Nevertheless, current NMR methodologies are limited by their ability to quantify metabolites present in low concentrations, and NMR-based metabolomics should be further developed to measure low-abundance metabolites in blood and tissues. In this context, it is noteworthy that an NMR spectroscopic approach with a new excitation pulse scheme, DREAMTIME, that improves sensitivity and detection limits in biological samples, has been presented recently [17]. Implementation and adoption of such advanced spectroscopic techniques to fecal and bio-fluid samples could enhance the power of NMR-based metabolomics in the field of food and nutrition science by allowing the detection of low-concentration metabolites including, for example, circulating levels of butyrate and propionate. 

The gut microbiome’s metabolism can basically be divided into saccharolytic and proteolytic activities, respectively. While saccharolytic activity as mirrored in the formation of SCFAs is desirable, proteolytic activity in the gut is often not desired as it may lead to the formation of toxic compounds [90,91]. High-protein diets have received increasing attention in dietary strategies focused on body weight management in recent years. In addition, a wealth of new protein sources (e.g., plants and insects) are currently being introduced as part of a focus on sustainable food production. However, protein quality and digestibility may vary. This results in a larger escape of protein residues to the large intestine, which may lead to the generation of amino acid-derivatives such as indoxyl sulfate, phenyl sulfate, and *p*-cresyl sulfate [92,93,94]. This is currently an issue that is rarely addressed, but it can be expected that metabolomics will be a key tool for obtaining in-depth knowledge on gut metabolites formed from proteolytic activity and thereby aid in a more complete assessment of new protein sources from a nutritional and health perspective.

Although many basic studies suggest beneficial effects of gut-modulating foods involved in immune modulation, evidence from clinical studies, especially randomized controlled trials, are sparse. Pronounced differences in gut microbial communities (e.g., *Firmicutes–Bacteroidetes* ratio and dominant bacteria) between laboratory animals and humans may result in significant differences in gut metabolism [95,96], which influence the translation from laboratory animals to humans. Thus, animal models should be considered a tool for obtaining mechanistic insight that subsequently must be backed up by validation in human studies.

## Figures and Tables

**Table 1:** Metabolite classes and metabolites in feces reported by NMR-based metabolomics.

Metabolite Class	Metabolites
Branched-chain amino acids	Leucine, Isoleucine, Valine
Aromatic amino acids	Phenylalanine, Tyrosine, Tryptophan
Other amino acids	Alanine, Arginine, Asparagine, Aspartate, Glutamate, Glutamine, Glycine, Histidine, Lysine, Methionine, Proline, Threonine, Sarcosine, Serine
Amino acid derivatives	p-cresol, 4-hydroxyphenyllactate, N-phenylacetylglycine, Acetoin, Creatine, Creatinine
Short-chain fatty acids	Acetate, Butyrate, Formate, Propionate, Butyrate, Isobutyrate, Valerate
Other organic acids	Bile acids, Caprylate, Citrate, Fumarate, Glycolate, Isocaproate, Lactate, Malate, Malonate, Phenylacetate, Pyruvate, Succinate, Taurine
Mono and disaccharides	Fructose, Glucose, Galactose, Lactose, Ribose, Xylose
Nucleotides and derivatives	Cytidine, Hypoxanthine, Thymine, Uracil, Xanthine
Alcohols	Glycerol, Ethanol, Methanol
Esters	2-methylbutyrate, 3-hydoxyphenlyacetate,4-hydroxyphenylacetate, Guanidoacetate, Methyl succinate
Alkaloids	Betaine, Carnitine, Choline, Dimethylamine, Ethanolamine,
Biogenic amines	Trimethylamine
Ketones	Acetone, Dihydroxyacetone

## Data Availability

Not applicable.
